# Characterization and commissioning of a Leksell Gamma Knife ICON system for framed and frameless stereotactic radiosurgery

**DOI:** 10.1002/acm2.13475

**Published:** 2022-01-22

**Authors:** Yue‐Houng Hu, Susannah V. Hickling, Jing Qian, C. Robert Blackwell, Luke B. McLemore, Erik J. Tryggestad

**Affiliations:** ^1^ Department of Radiation Oncology Division of Medical Physics Mayo Clinic Rochester Minnesota

**Keywords:** CBCT, end‐to‐end testing, film dosimetry, Gamma Knife IU, SRS

## Abstract

**Purpose:**

The Leksell Gamma Knife Icon unit (IU) was introduced recently as an upgrade to the Perfexion unit (PU) at our Gamma Knife practice. In the current study, we sought mainly to characterize dosimetry and targeting accuracy of the IU treatment deliveries using both invasive frame and frameless treatment workflows.

**Methods:**

Relative output factors were measured by delivering single‐shot 4, 8 and 16 mm radiation profiles in the manufacturer's acrylonitrile butadiene styrene spherical phantom in coronal and sagittal planes using EBT3 film. Resultant dosimetry was compared with the manufacturer's dose calculation and derived output factors were compared with the manufacturer's published value. Geometric consistency of stereotactic coordinates based on cone‐beam computed tomography (CBCT) versus the traditional conventional CT‐based method was characterized using a rigid phantom containing nine fiducial indicators over four separate trials. End‐to‐end (E2E) testing using EBT3 film was designed to evaluate both dosimetric and geometric accuracy for hypothetical framed and frameless workflows.

**Results:**

Relative output factors as measured by the manufacturer were independently confirmed using EBT3 film measurements to within 2%. The mean 3D radial discrepancy in stereotactic space between CBCT and CT‐based definition over the sampled locations in our rigid geometry phantom was demonstrated to be between 0.40 mm and 0.56 mm over the set of trials, larger than prior reported values. E2E performed in 2D demonstrates sub‐mm (and typically < 0.5 mm) accuracy for framed and frameless workflows; geometric accuracy of framed treatments using CBCT‐defined stereotactic coordinates was shown to be slightly improved in comparison with those defined using conventional CT. Furthermore, in phantom, frameless workflows exhibited better accuracy than framed workflows for fractionated treatments, despite large magnitudes of introduced interfraction setup error. Accuracy of dosimetric delivery was confirmed in terms of qualitative comparisons of dose profiles and in terms of 2D gamma pass rates based on 1%/1 mm criteria.

**Conclusion:**

The IU was commissioned for clinical use of frameless and framed treatment protocols. The present study outlines an extensive E2E methodology for confirmation of dosimetric and geometric treatment accuracy.

## 1 INTRODUCTION

The Leksell Gamma Knife® Icon Unit (IU) was developed as an update to the previous Perfexion Unit (PU) model, implementing cone‐beam computed tomography (CBCT) and intra‐fraction motion management (IFMM) systems. As with the PU, the IU radiative system consists of 192 sealed ^60^Co sources, distributed spatially into eight motorized sectors. To deliver treatment, sectors are translated within the IU bore over a circular tungsten collimator, oriented to deliver radiation to a single radiative focus. Available collimation includes apertures of 4, 8, and 16 mm diameter; each sector can deliver a single aperture size (including “sector off”) per radiation “shot” location.

Numerous prior studies on the IU have been conducted, including general descriptions of commissioning experiences,^1^ assessment of the accuracy^2^, ^3^ and quality assurance^4^ (QA) of the image guidance system. Past studies on the previous PU model have assessed the stability of the definition of stereotactic coordinates using a stereotactic head frame,^5^ commissioning and QA of the automatic positioning system,^6^ QA of the PU beam accuracy,^7^, ^8^ and evaluation of the dose calculation software^9^ and a secondary dose calculation system.^10^ Prior studies have disclosed early clinical experiences regarding frameless workflows^11^, ^12^ as well as predictors of treatment interruption^13^ using frameless processes. However, to date, no studies have outlined commissioning experience for frameless treatment deliveries. The present study describes the most relevant aspects of our practice's experience in characterizing and commissioning a newly installed IU system for use in both framed and frameless treatment contexts.

## 2 METHODS

### 2.1 Dose verification

#### 2.1.1 Independent determination of effective output factors

To ensure dosimetric accuracy during treatment measurement of effective output factors (OF_eff_) as a function of shot collimation was independently confirmed via relative measurements using EBT3 film. Film was placed in the center of the vendor‐provided acrylonitrile butadiene styrene (ABS) spherical dosimetry phantom (SDP). The ABSSDP was chosen because it can be converted for film use with relative ease in comparison to the solid water SDP. Further, the ABSSDP allowed for both axial and coronal film orientations. The ABSSDP was indexed to the patient positioning system (PPS) using the vendor‐provided jig, allowing for phantom coincidence with the IU focus, defined at *x‐*, *y‐*, and *z‐*positions of 100, 100, 100 mm in stereotactic coordinate space.

OF_eff_ measurements were performed using films oriented axially. One‐minute plans were delivered to IU focus with all sectors open for each of the given collimator sizes. Film was scanned at 200 dpi and analyzed in the FilmQA Pro software (Ashland, Bridgewater, NJ, USA) following a previously published film dosimetry protocol^14^ involving only the red channel and incorporating a 4 Gy reference strip. Circular regions of interest (ROI) of 4, 2.5, and 1.4 mm diameter were used to determine dose delivered by the 16, 8, and 4 mm collimator plans respectively. Measurements were repeated 5 times for each collimator size and average dose values were normalized to the result obtained for the 16 mm collimator.

#### 2.1.2 Dose profile measurements

Using the same experimental setup described for OF measurement, EBT3 film measurements were additionally performed in the coronal orientation. One representative axial film per collimator was arbitrarily chosen from the OF measurements above for dose profile verification. Measured 2D dose distributions in both the coronal and axial planes were then compared to treatment planning system (Leksell Gamma Plan version 11.1.1: LGP) calculated planar dose distributions; 1D dose profiles were also generated. Note that all LGP dose calculations assume an all‐water medium and were executed using the TMR10 algorithm. Dose matrices were sampled isotropically at a resolution of 0.5 mm and exported to Aria (the institutional record and verify system). Each dose plane was then selected by aligning fiducial markers in the phantom insert and exported to a 350 × 350 matrix at a resolution of 0.17 mm per pixel (i.e. 60 × 60 mm^2^) resulting in resampling of the grid, potentially obliquely with respect to the treated stereotactic coordinate system. Registration between measurement and planned doses was performed using the algorithm in Film QA Pro, which optimizes translations and rotations to maximize gamma pass rates.

### 2.2 Positional integrity

#### 2.2.1 Stereotactic coordinates from on‐board CBCT versus conventional CT with fiducial indicator box

**FIGURE 1:**
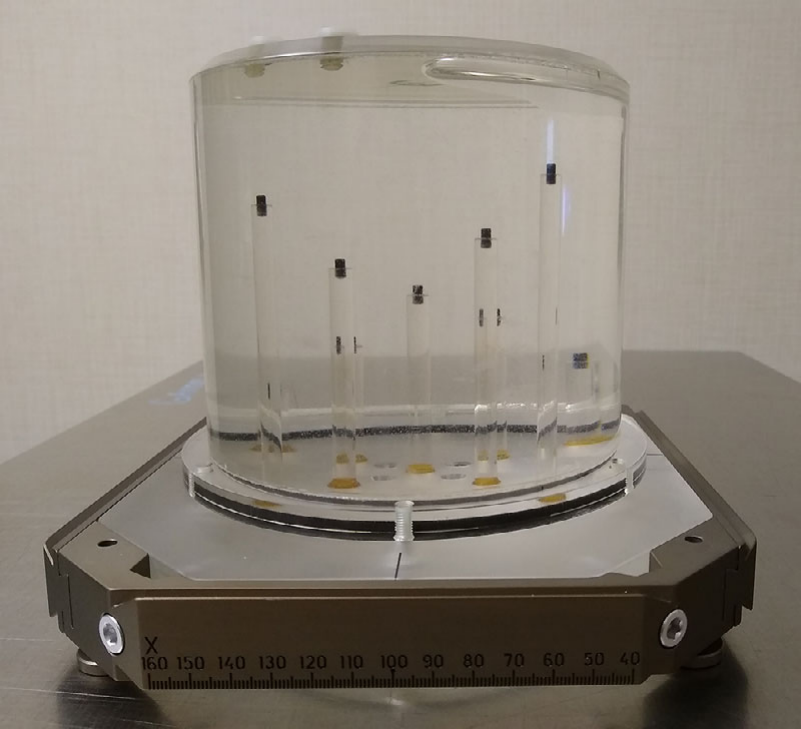
Photograph of the in‐house candlestick phantom

Stereotactic coordinates defined by CT or magnetic resonance imaging (MRI) via fiducial marker (from the given fiducial indicator box) has long been the gold standard for Gamma Knife treatment planning. For the IU, stereotactic coordinates may be alternatively defined by on‐board CBCT. It is therefore imperative to evaluate the consistency between these two methods of stereotaxy. To this end, we employed an in‐house designed and fabricated GK‐dedicated cylindrical imaging phantom (heretofore referred to as the “candlestick phantom”) containing nine imaging markers (Figure 1). The phantom was built in collaboration with the institutional Division of Engineering. The candlestick phantom is water‐filled and has nine posts, each of differing length and equipped with a fiducial marker tip providing CT contrast for identification. The assembly is permanently indexed within a Leksell model G‐Frame and can therefore accept the G‐Frame adaptor for fixation.

The phantom was mounted in a fiducial indicator box and simulated on a clinical CT scanner. CBCT imaging was performed with the phantom mounted on the IU PPS using both available (vendor‐defined) high and low dose CBCT protocols (CTDI 6.3 and 2.5 mGy, respectively). In LGP, stereotactic coordinates were defined on CT simulation scans using the fiducial indicator box. Stereotactic coordinates of the fiducial tips were extracted based on manual placement of the image navigation crosshairs in three orthogonal cut planes. The reproduction of stereotactic coordinates as defined by each CBCT protocol in comparison to diagnostic CT was analyzed. It should be noted that two separate CT fiducial boxes were used to define stereotactic coordinates on conventional CT images.

### 2.3 End‐to‐end (E2E) testing and IU workflows

**FIGURE 2:**
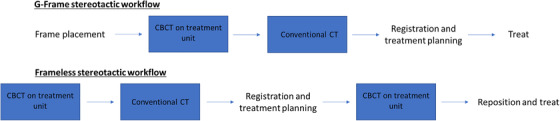
Flowcharts of hypothetical G‐Frame (top) and frameless (bottom) stereotactic workflows evaluated

**FIGURE 3:**
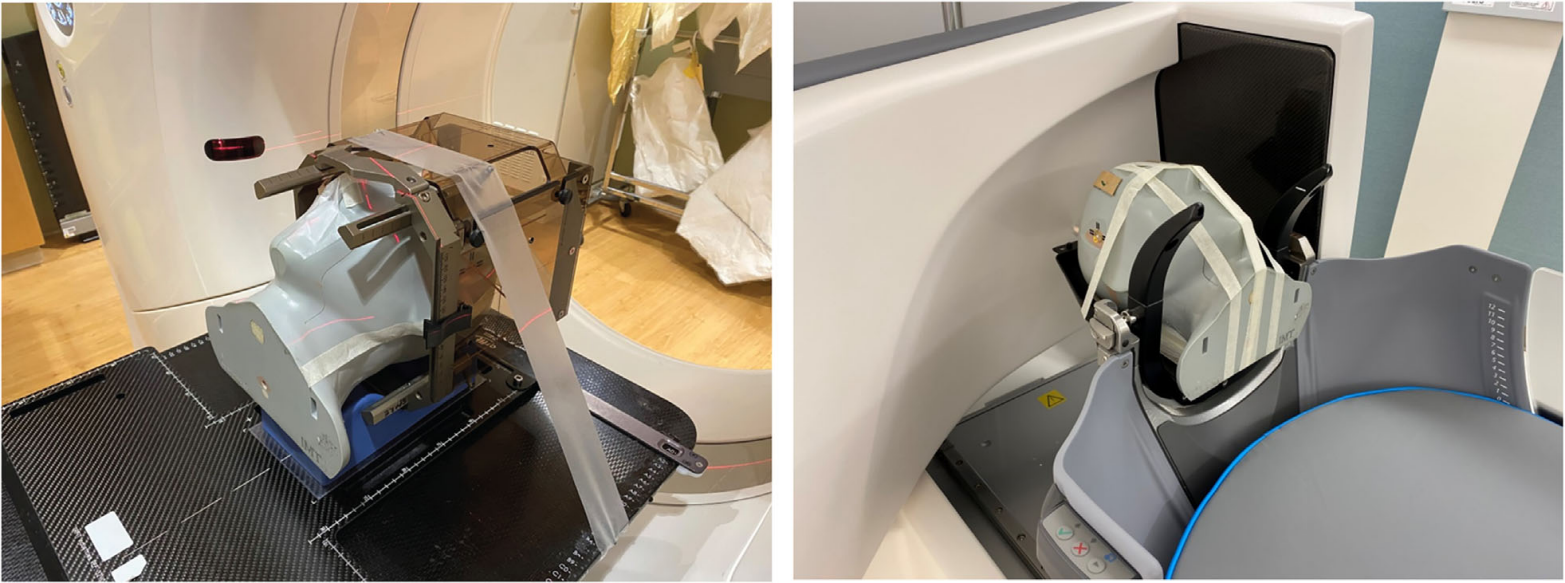
Photos of tested G‐Frame (left) and frameless (right) setups for E2E testing

E2E testing was performed using the MAX‐HD End‐to‐End SRS phantom (Integrated Medical Technologies, Inc., Troy, NY). This phantom includes three cubical target inserts (with visible target CT‐number heterogeneity), each of which may be bisected for film inclusion. Target inserts measured 4 cm^3^ (TG_S_), 5 cm^3^ (TG_M_), and 6 cm^3^ (TG_L_) in size. The hypothetical workflows for both G‐Frame (framed) and frameless deliveries that were evaluated are depicted in Figure 2. Photos of the treatment setup for each workflow may be seen in Figure 3. Given the choice of anatomic phantom, conventional CT was employed as the reference diagnostic image set. It should be noted that the IU unit and planning system allows flexibility in terms of a given institution's clinical workflow. At minimum, one stereotactic image set is needed, whether provided by on‐board CBCT or from traditional fiducial indicator box‐derived coordinate definition for framed workflows. Diagnostic imaging need not follow acquisition of initial CBCT but may be taken prior to G‐Frame fixation or frameless mask creation, since anatomical fusion with any stereotactically defined planning image is possible. For framed cases on the IU, a CBCT may be acquired immediately pretreatment to verify targeting accuracy. If unacceptable deviations are observed the plan must be replanned using the latest CBCT image as the stereotactic reference. Framed treatments do not require acquisition of a CBCT: PU‐style G‐Frame fixation using only traditional indicator‐box‐based coordinate definition/targeting remains possible.

#### 2.3.1 Verification of end‐to‐end geometric accuracy

End‐to‐end (E2E) shot accuracy was tested for both the hypothetical framed and frameless workflows described in section 2.3. Fiducial markers (steel shards, well under 0.5 mm, hand‐cut from either paper staples or paper clips) were affixed with tape arbitrarily to films cut to fit in TG_S_, TG_M_, and TG_L_. These targets were then loaded into the MAX‐HD phantom in either sagittal or coronal orientation. An individual 4 mm shot was centered on each imaging‐resolved (human interpreted) fiducial marker location and planned for delivery of 4 Gy to the 50% isodose line. The phantom was then set up (Figure 3) and the plan delivered according to the tested protocol. For the hypothetical framed workflow, 2D (in film‐plane) geometric accuracy of treatment delivery may be evaluated for both conventional CT‐defined (via fiducial indicator box) and on‐board CBCT‐defined stereotactic shot coordinates.

Films were scanned at a resolution of 0.127 mm per pixel (200 dpi). Analysis was performed by finding the centroid of the exposed shots. Briefly, all images were imported and analyzed in Matlab (Mathworks, Natick, MA) based on scanned pixel values. Images were converted to a binary mask by employing the im2bw function. Pixels corresponding to scanned intensity values below 40% of the maximum value were set to 0 (e.g. exposed areas) with all other pixels set to 1. These binarized images were morphologically closed, inverted, and flood‐filled by employing packaged Matlab algorithms (imclose, imcomplement, and imfill, respectively) included in the image processing toolbox. Centroids were calculated as the center‐of‐mass of each binarized, morphologically closed, inverted, and flood‐filled spot. The position of each centroid was compared to the human‐observed position of each metal shard.

#### 2.3.2 Verification of E2E absolute dose accuracy

For testing of absolute dosimetry of plan delivery, a two‐target plan was designed for the MAX‐HD phantom to treat resolvable phantom targets within the TG_L_ and TG_S_ inserts. Sagittal film orientation was used for all trials. Planned prescription dosage was selected to optimize EBT3 accuracy and was not intended to represent clinically meaningful treatment times.

Planar dose delivery was evaluated using the same method described in section 2.1.2. Measured dose distributions were compared to LGP calculations via gamma analysis with a minimum dose threshold of 10% and a pass criterion of 1%/1 mm.^15^

**FIGURE 4:**
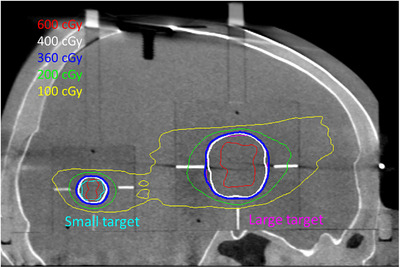
Planned dose distribution on the stereotactic CBCT for G‐Frame E2E testing for the large and small targets

For E2E testing of the framed workflow, the large lesion was prescribed 3.6 Gy to the 45% isodose line and treated with 17 shots. The small lesion was prescribed 4 Gy to the 50% isodose line and treated with three shots. Figure 4 shows the dose distribution on a sagittal slice of the initial CBCT used to define the stereotactic frame of reference.

For E2E testing of the frameless workflow a five‐fraction plan was developed. The large lesion was treated with 18 shots to a dose of 20 Gy prescribed to the 50% isodose line. The small lesion was prescribed 18 Gy to the 45% isodose line and treated with 10 shots. The plan was delivered for four of five planned fractions, with the phantom repositioned between each fraction, in some cases introducing large, clinically unrealistic perturbations. Per the frameless workflow in Figure 2, a CBCT was acquired immediately prior to treatment for each fraction and registered to the stereotactic coordinate system. The translations and rotations required by rigid CBCT registration were recorded. Dose on the pretreatment CBCT was reviewed prior to delivery.

## 3 RESULTS

### 3.1 Dose verification

#### 3.1.1 Measurement of dose‐to‐water and output factors

Measurements of OF_eff_ may be found in Table 1. Good agreement is observed for all collimator sizes. Uncertainties in film measurements were defined as equal to two standard deviations calculated over five film measurements for each collimator size.

**TABLE 1:** Comparison of manufacturer and film measured output factors

Collimator size (mm)	Manufacturer output factor	Film measured output factor
16	1.000	1.000 ± 0.02
8	0.900	0.900 ± 0.03
4	0.814	0.830 ± 0.02

#### 3.1.2 Dose profile measurements

**FIGURE 5:**
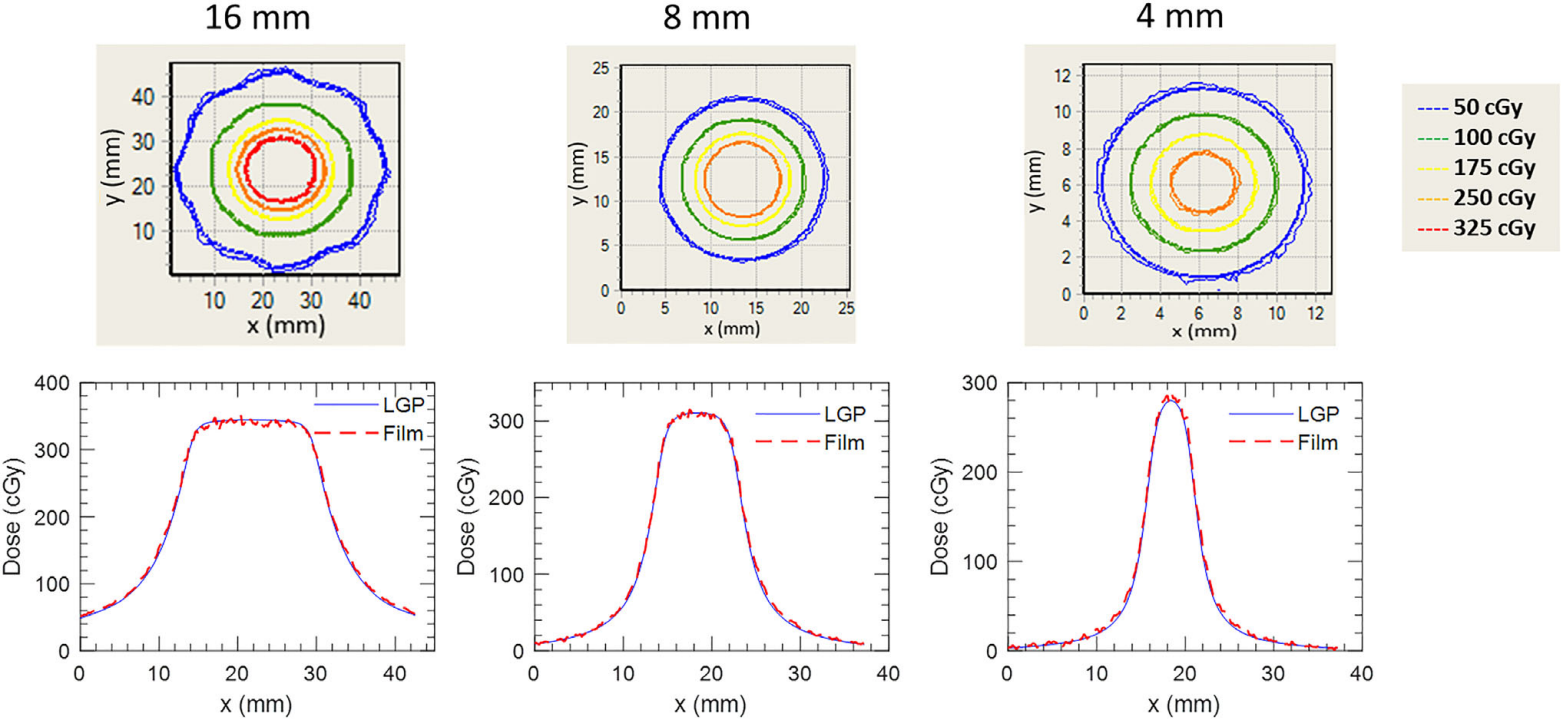
Isodose and right to left profile comparisons between film and LGP measured dose distributions at different collimator sizes for axial plane measurements in the spherical water phantom.

**FIGURE 6:**
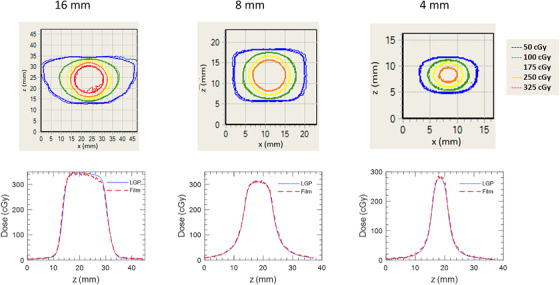
Isodose and superior to inferior profile comparisons between film and LGP measured dose distributions at different collimator sizes for coronal plane measurements in the spherical water phantom

Displayed in Figure 5 is a comparison between axial planar film measurements and corresponding LGP calculations for dose distributions delivered by one of the 16 mm (left), 8 mm (right), or 4 mm (right) collimators to the ABSSDP. Figure 6 depicts the same results for a coronal plane. Qualitative inspection demonstrates excellent agreement in both 2D isodose and 1D dose profile comparisons. Quantitative analysis in terms of gamma pass rates for both axial and coronal film orientations are tabulated in Table 2. Pass rates exceeded 99.8% in 5 of 6 cases with the one exception being the 4 mm coronal measurement (98.5%).

**TABLE 2:** Gamma pass rates (1%/1 mm criterion with 10% dose threshold) for film measurements in comparison to LGP plan

	Gamma pass rates (1%/1 mm, 10% threshold)	
Collimator size (mm)	Axial	Coronal
16	99.99	99.81
8	99.97	99.96
4	99.85	98.54

### 3.2 Positional integrity

#### 3.2.1 Stereotactic coordinates from CBCT versus CT with fiducial indicator box

Maximum differences in stereotactic coordinates defined from conventional CT imaging by using either fiducial box was quantified at 0.4 mm with the mean disagreement being 0.04 mm. The total uncertainty of fiducial positions (defined as two standard deviations from the mean value of a given fiducial marker coordinate in any Cartesian direction) found by conventional CT was equal to 0.10 mm. Using the mean fiducial positions derived from conventional CT data as the ground truth, deviations (*d*) in each Cartesian direction and radially (*r*) for each CBCT protocol set are tabulated in Table 3. The maximum value of *d_r_* for the lower and higher dose CBCT settings (CTDI_2.5_ and CTDI_6.3_, respectively) images were 0.63 and 0.70 mm, respectively, with mean *d_r_* being quantified at 0.44 and 0.46 mm.

**TABLE 3:** Deviations (*d*) in each Cartesian coordinate (*x*, *y*, and *z*) and radially (*r*) for stereotactic coordinates for fiducial markers using CTDI_2.5_ and CTDI_6.3_ CBCT in comparison with conventional CT

	CTDI_2.5_				CTDI_6.3_			
Fiducial	*d_x_*	*d_y_*	*d_z_*	*d_r_*	*d_x_*	*d_y_*	*d_z_*	*d_r_*
1	0.60	–0.10	–0.15	0.63	0.60	–0.10	–0.35	0.70
2	0.35	–0.05	0.30	0.46	0.45	–0.05	0.30	0.54
3	0.55	–0.15	–0.05	0.57	0.45	–0.15	–0.05	0.48
4	0.50	–0.05	0.15	0.52	0.50	–0.05	0.15	0.52
5	0.50	–0.10	0.00	0.51	0.50	0.00	–0.10	0.51
6	0.25	–0.15	–0.05	0.30	0.25	–0.15	0.05	0.30
7	0.25	0.00	0.20	0.32	0.35	0.00	0.20	0.40
8	0.35	–0.15	–0.05	0.38	0.25	–0.05	0.15	0.30
9	0.25	0.15	0.05	0.30	0.25	0.25	–0.05	0.36

### 3.3 End‐to‐end testing

#### 3.3.1 Verification of end‐to‐end geometric accuracy

**FIGURE 7:**
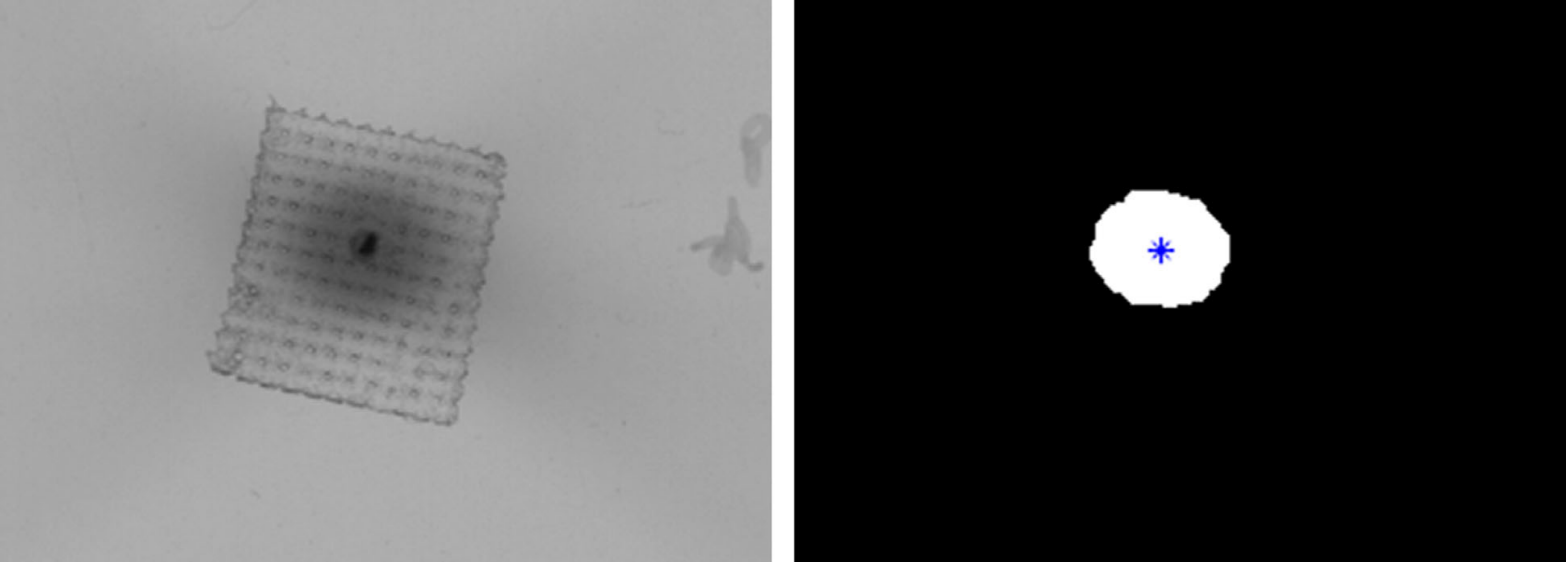
Scanned film (left) and binarized (right) images for a single shot of the E2E test. The metal shard is observable as the most radioopaque region of the film at the center of the shot. The centroid of the binarized image is depicted by the star

For the G‐frame workflow depicted in Figure 2, comparisons between shot centroid and observed fiducial location for both diagnostic CT‐ and CBCT‐defined stereotactic shot coordinates are tabulated in Table 4. An example of a scanned film and its corresponding binarized image may be seen in Figure 7. The star in the binarized image denotes the centroid of the binarized dose distribution. Note that the metal shard is clearly identifiable in the scanned film image. Stereotactic shot coordinates defined by either modality resulted in a maximum 2D‐radial deviation of fiducial and shot centroid of 0.524 mm. Mean deviations for CBCT‐defined and CT‐defined coordinate systems were 0.165 and 0.285 mm, respectively.

**TABLE 4:** Comparison of 2D (in plane) radial geometric error (in mm) in shot placement for E2E testing of the IU using both CT‐ and CBCT‐defined stereotactic shot coordinates for TG_L_, TG_M_, and TG_S_ inserts. These trials were carried using only the coronal film orientation using a framed workflow

	CT‐defined			CBCT‐defined		
Shard	TG_L_	TG_M_	TG_S_	TG_L_	TG_M_	TG_S_
1	0.127	0.127	0.284	0.127	0.381	0.000
2	0.458	0.284	–	0.180	–	–
3	0.458	–	–	0.180	–	–
4	0.254	–	–	0.127	–	–

Similarly, the comparison of 2D E2E targeting error for the framed workflow versus the frameless workflow is tabulated in Table 5. For these trials, CBCT was used to defined stereotactic coordinates and films were oriented both coronally and sagittally. The maximum 2D radial errors observed were 0.718 and 0.284 mm for framed and frameless treatment deliveries, with mean values being 0.429 and 0.225 mm, respectively.

**TABLE 5:** Comparison of 2D radial geometric error for framed vs. frameless workflows, both using CBCT‐defined stereotactic coordinates. TG_L_ was oriented in the coronal plane with TG_S_ in the sagittal

	G‐Frame workflow		Frameless workflow	
Shard	TG_L_	TG_S_	TG_L_	TG_S_
1	0.284	0.284	0.284	0.180
2	0.718	–	0.180	–

#### 3.3.2 Verification of end‐to‐end absolute dose distributions

**FIGURE 8:**
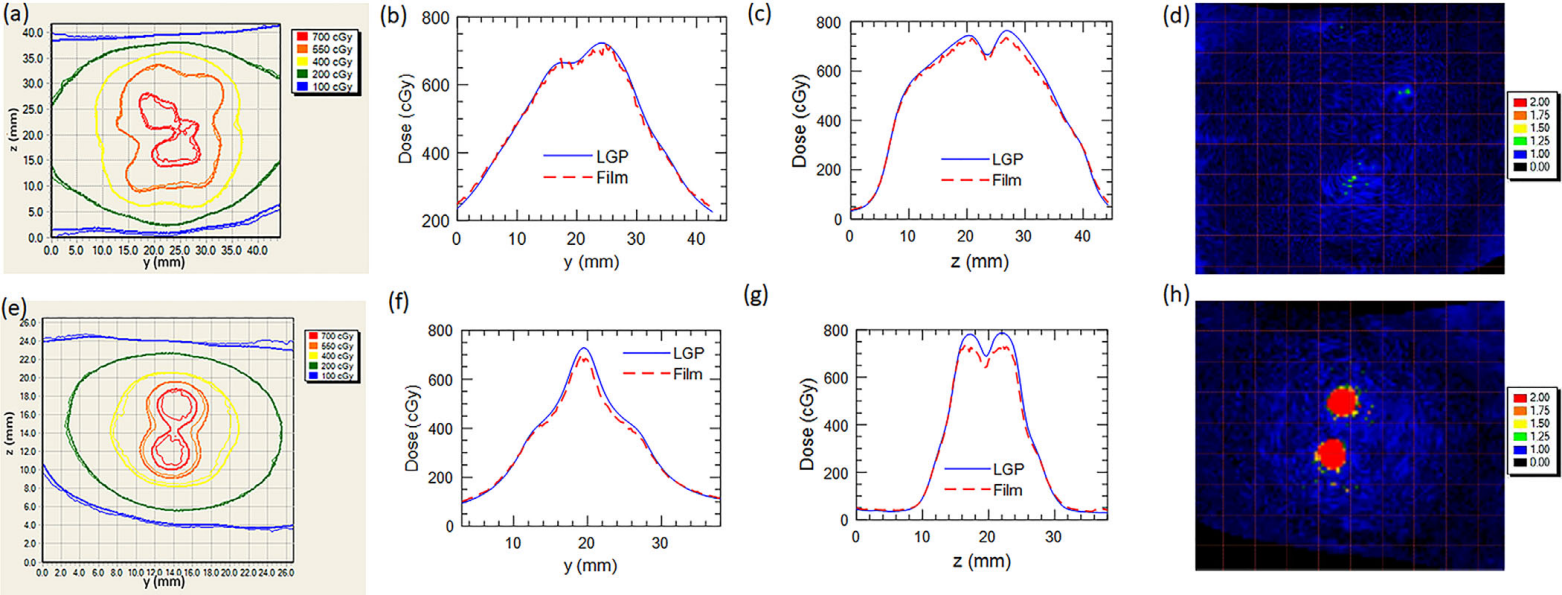
Comparison of isodose lines (a); AP profiles (b); SI profiles (c); and gamma maps (d) for framed E2E dose distributions for the sagittal plane measured through the large target. Comparison of isodose lines (e); AP profiles (f); SI profiles (g); and gamma maps (h) for framed E2E dose distributions for the sagittal plane measured through the small target. In the isodose plots, thick lines are LGP isodose lines while the thin lines correspond to film

Figure 8 plots a comparison of the film measured and LGP calculated dose distributions for the large and small targets for the framed case. Good agreement is observed with 1%/1 mm gamma pass rates of 99.7% and 97.1% for the small and large targets respectively.

**FIGURE 9:**
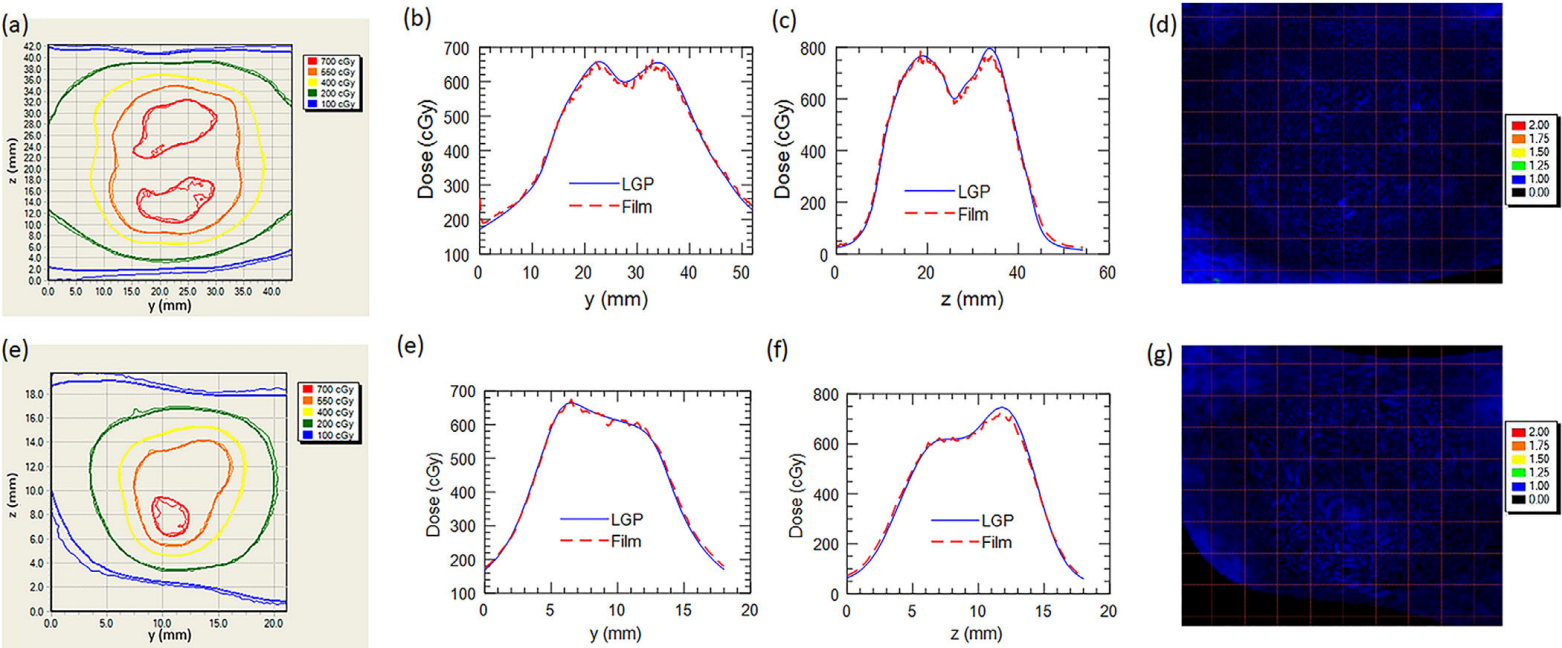
Comparison of isodose lines (a); AP profiles (b); SI profiles (c); and gamma maps (d) for frameless dose distributions for the sagittal plane measured through the large target. Comparison of isodose lines (e); AP profiles (f); SI profiles (g); and gamma maps (h) for frameless dose distributions for the sagittal plane measured through the small target. In the isodose plots, thick lines are LGP isodose lines while the thin lines correspond to film

Figure 9 plots comparisons of the film‐measured and LGP‐calculated dose distributions for fraction 1 of the frameless E2E treatment of the large and small target. The result is representative of those from the subsequent fractions. The 1%/1 mm gamma pass rates resulting from this analysis are provided in Table 6. For reference, Table 6 also lists the translations and rotations introduced by the registration prior to the delivery of each fraction of the frameless E2E testing. Note that delivery of fraction four was intentionally halted partially through treatment and the phantom repositioned. The delivery of fraction four was subsequently stopped by the IFMM due to inadvertent phantom slippage; delivery was resumed after reacquisition of the CBCT and reregistration against the planning reference CBCT. Note that the IFMM was independently tested as part of our institution‐specific IU unit commissioning but results are not reported due to practical constraints on manuscript length.

**TABLE 6:** Registration‐introduced translation and rotations and gamma pass rates of dose delivery for the 4‐fraction frameless treatment

	Translation (mm)			Rotation (^o^)			Gamma pass rates (1%/1 mm)	
Fraction	∆*X*	∆*Y*	∆*Z*	∆*X*	∆*Y*	∆*Z*	Large	Small
1	5.01	2.68	10.30	0.34	–2.07	–2.58	99.8	100
2	0.21	1.48	37.83	3.03	0.42	–6.13	97.9	97.8
3	–1.81	4.63	–5.17	0.02	–0.49	2.76	99.3	97.1
4	0.81	1.75	21.25	1.37	–1.56	0.16	98.9	99.7
4 (repositioned)	–1.28	–0.04	44.55	4.34	–4.62	–2.99	–	–
4 (halted)	–0.57	–0.15	36.45	0.3	0.27	–0.83	–	–

## 4 DISCUSSION

### 4.1 Output verification

In‐house film measurements of OF_eff_ agreed well with acceptance testing with deviations being observed for the smallest collimator size. Observed discrepancies using film measurements may be affected by the choice of ROI over which output was defined. Prior studies that have compared user measurements of OF_eff_ to those measured by the manufacturer found accuracies ranging between 0.5% and 5% using film dosimetry^16^, ^17^ and between 0.1% and 3.33% using polymer gel dosimeters.^18^ The reported result (deviations of 0% and 1.93% for 8 and 4 mm collimator sizes, respectively) of the present manuscript represents an excellent agreement with vendor specifications.

### 4.2 Stereotactic coordinates from CBCT versus CT with fiducial indicator box

Measurements conducted using the candlestick phantom confirm that the coordinate system defined by CBCT is consistent with that defined from simulation CT with localizer box. It should be noted that disagreements in coordinate definition are larger than those reported in prior literature.^1^ Increased uncertainties may be driven by imaging noise precluding precise identification of marker tips. Inaccuracies associated with the integrity of the fiducial indicator box (its construction and its seating on the G‐Frame) as well as flexion of the G‐Frame are convolved into this finding.

### 4.3 Verification of end‐to‐end accuracy

2D geometric accuracy of spot delivery based on CT or CBCT was quantified in a rigid anthropomorphic phantom using planar film measurements to be within 0.8 mm for all framed delivery trials, with CBCT‐defined coordinates exhibiting lower deviation between shot centroids and fiducial markers when compared to CT‐defined coordinates. Using a frameless workflow, the observed 2D deviations were below 0.3 mm in all cases. We reported that mean 2D deviations were below 0.5 and 0.3 mm for framed and frameless treatment deliveries. When a head frame was employed, localization was found to be most accurate when CBCT was registered to stereotactic coordinates based on diagnostic quality CT imaging. The slight improvement in delivery accuracy for frameless workflows we observed may be attributed to automatic adjustment of plan to the delivery setup (CBCT‐guidance) and an overall error reduction compared to the G‐frame workflow (without sources of uncertainty associated with the G‐Frame and fiducial box‐based coordinate inference). This finding does not necessarily imply that frameless IU treatments are more accurate that G‐framed treatments in a clinical context. It should be noted that underlying uncertainties in the definition of the fiducial marker location both in treatment planning and in data analysis exist. Given the size of the shards (well under 0.5 mm) in context of the CT or CBCT imaging resolution, this amounts to a potential measurement error of approximately ±0.5 mm.

Use of MRI in the planning and targeting process is expected to increase overall delivery uncertainty due to MRI image distortion: a complex problem given the dependency of MRI distortion on the MRI hardware, the particular sequence and reconstruction employed and patient‐specific issues such as magnetic susceptibility and chemical shift. Thus, E2E measurements in MRI phantoms cannot be relied upon to be representative of actual patient treatments. To address this potential gap, our commissioning process involved imaging both MRI and CT fiducial localizer boxes with CT and performing image registration to (semiquantitatively) confirm geometric integrity of the MRI localizer boxes.

Dosimetric accuracy in the anthropomorphic phantom was also confirmed for both framed and frameless deliveries. Gamma pass rates were above 97% using a 1%/1 mm criterion for all testing conditions. However, discrepancies appeared in the high‐dose regions of the framed, small‐target treatment. Using a 2%/1 mm or a 4%/1 mm criterion improves pass rates to greater than 98% and 99%, respectively. These failing regions were the result of a lack of uniformity in the reference film exposure and potentially also due to the resampling process prior to dose plane extraction noted in section 2.3.1. Given both the absence of signal uniformity that the ROI used to define the reference dose (chosen to reside toward the center of the film) was observed to be in an erroneously high‐intensity area of the film resulted in dose maps used to perform the comparison with LGP being uniformly cold. Further, the selection of dose planes for film analysis, potentially in orientations oblique to the stereotactic coordinate system, may also introduce small spatial uncertainties, which may result in substantial dose errors during gamma analysis. The possibility of real delivery errors was dismissed due to (1) confirmation of dosimetric accuracy in all other film measurements, including those not published in the present manuscript; (2) confirmation of dosimetric accuracy via ionization chamber measurements in commissioning and regular machine QA; and (3) improvement of gamma pass rate (greater than 98% at 1%/1 mm and greater than 99% at 2%/1 mm) when a more appropriate reference film ROI is used.

In the frameless study we introduced large and rather unrealistic translations up to 4.5 cm and rotations over 6^o^. Nevertheless, the IU CBCT‐guidance system and plan adaptation process allowed for dosimetric recapitulation of the intended dose: 2D dosimetric gamma pass rates exceeding 97% were achieved (1%/1 mm) for the four fractions simulated. Translations and rotations within a clinical setting are expected to be much less than those tested here. Lower isodose lines exhibit the largest disagreement for the frameless E2E delivery, corresponding to applied rotations and due to the lack of 6DOF corrections with the PPS. It must also be noted that agreement of dosimetric accuracy was found to be best around the 4 Gy isodose line, corresponding to the dose of the delivered reference strip in the EBT3 scanning and calibration protocol, which was an expected finding. Thus, disagreement observed at higher and lower doses may not necessarily be indicative of actual dosimetric error but instead be due to limitations associated with use of EBT3 film.

## 5 CONCLUSIONS

The present study outlines the commissioning experience of the Gamma Knife IU for both framed and frameless treatment paradigms. It further presents a novel methodology for characterization of the Gamma Knife IU clinical performance employing extensive E2E testing. Both protocols, when delivered to an anthropomorphic phantom exhibit good agreement between expected and delivered radiation dose in terms of geometric accuracy and relative and absolute dosimetry.

## CONFLICT OF INTEREST

The authors claim no conflicts of interest.

## AUTHOR CONTRIBUTIONS

All authors substantially contributed to the experimental design and acquisition of data for the submitted study. Principle data analysis were performed by Yue‐Houng Hu, Susannah V. Hickling, and Jing Qian. Primary composition of the manuscript was performed by Yue‐Houng Hu. Finally, all authors contributed materially to the content editing process.
